# “A year-long, fortnightly, observational survey in three European countries of patients with respiratory allergies induced by house dust mites: Methodology, demographics and clinical characteristics”

**DOI:** 10.1186/s12890-016-0246-9

**Published:** 2016-05-23

**Authors:** Pascal Demoly, Andrea Matucci, Oliviero Rossi, Carmen Vidal

**Affiliations:** Allergy Division, Pulmonology Department, Hôpital Arnaud de Villeneuve, University Hospital of Montpellier, Montpellier, France; Sorbonne Universités, UPMC Paris 06, UMR-S 1136 INSERM, IPLESP, Equipe EPAR, Paris, France; Department of Internal Medicine, Section of Immunoallergology and Respiratory Diseases, University of Florence, Florence, Italy; Allergy Service, Complejo Hospitalario Universitario de Santiago, Santiago de Compostela, Spain

**Keywords:** Respiratory allergy, House dust mite, Long-term observational survey

## Abstract

**Background:**

House dust mite (HDM) allergens constitute the leading trigger for the symptoms of persistent respiratory allergies (such as allergic rhinitis and asthma). However, the fluctuating, pernicious nature of the symptoms has given rise to a perception that HDM-induced respiratory allergy is not a “real” disease.

**Methods:**

In order to assess the self-reported disease profile and behaviour of adult patients with a self-reported history of severe, poorly controlled, physician-diagnosed HDM respiratory allergy, we performed an observational, international, multicentre survey in three European countries (France, Italy and Spain). Participants were included in the survey if they passed a short Internet-based screening questionnaire. Following completion of a detailed post-inclusion questionnaire, 28 fortnightly telephone interviews were used to gather extensive data on the participants’ symptom prevalence and intensity, medical consultations, disease burden, quality of life, and medication use from late May 2012 to early July 2013.

**Results:**

Twenty-two thousand nine hundred ninety five individuals completed the Internet screening questionnaire and 339 respondents (67 % female) met all the inclusion criteria. 313 of the 339 (92 %) completed the post-inclusion questionnaire (*n* = 114 in Italy, 92 in France and 107 in Spain). The median time since the first symptoms of HDM allergy was over 13 years in all three countries. The response rate for the fortnightly interviews averaged 75 % (range: 29 to 97 %). The reported fortnightly prevalence of nasal and ocular symptoms peaked in the autumn (September to November) and spring (March to May). These peaks in prevalence coincided with increased reports of symptom worsening and higher physician consultation rates. In participants not allergic to pollen, the autumn and spring peaks were accompanied by a third peak in late December 2012. Very few participants reported that their symptoms had never improved (4 %) or never worsened (11 %) during the survey period.

**Conclusions:**

In a survey in France, Italy and Spain, patients with severe HDM-induced respiratory allergies experienced a complex set of changing, troublesome symptoms throughout the year, with peaks in spring, autumn and (to a lesser extent) mid-winter.

**Electronic supplementary material:**

The online version of this article (doi:10.1186/s12890-016-0246-9) contains supplementary material, which is available to authorized users.

## Background

House dust mites (HDMs) constitute a major, persistent source of indoor aeroallergens and constitute the leading cause of respiratory allergies such as allergic rhinitis (AR) and allergic asthma. These conditions affect more than 500 million people worldwide [1, 2]; albeit with significant geographical variations (i) in the prevalence and types of HDMs [1], (ii) domestic levels of HDM allergens [3] and (iii) levels of sensitization to HDM allergens in the general population [4, 5]. The link between AR and asthma is well established, and exposure to HDM allergens early in life is associated with an increased risk of asthma [6, 7].

Patients consulting an allerggist for HDM allergy tend to have moderate-to-severe, persistent disease profiles. In a French, retrospective survey of 1289 patients in whom allergy immunotherapy (AIT) had been initiated, Trebuchon et al. [8] reported that 64.9 % had a history of moderate-to-severe, persistent AR and that 50 % also suffered from (mainly mild, intermittent) asthma. Furthermore, 62.5 % of the patients were polysensitized and thus potentially polyallergic (meaning those polysensitized patients with corresponding symptoms related to those allergens to which they show sIgE). Indeed, 22 % of the patients had initiated AIT for a second allergen (usually grass pollen). This profile was seen in both adults and children [8, 9]. The complexity of clinical profiles in HDM allergy explains why Worm et al. [10] found that 88 % of 5751 HDM-allergic patients surveyed in Germany had consulted a specialist physician rather than a general practitioner; this contrasted with the patients with grass pollen allergy only, most of whom were treated in general practice. The treatment profile for HDM-allergic patients also differed, with significantly greater used of topical corticosteroids. The average number of co-prescriptions was 2.1, and around 9 % of the patients had received a prescription for AIT [10, 11].

The symptoms of HDM-induced allergy can have a significant negative impact on a patient’s quality of life (QoL) [12]. Although the harmful symptoms related to HDM-induced respiratory allergy are usually always present to some extent, their intensity varies over time as domestic HDM populations and allergen levels fall or rise as a function of weather-related factors or other changes in the indoor living environment [13, 14]. The pernicious nature of persistent, HDM-induced allergy has given rise to the perception among patients [12] that it is not a “real” disease. For example, sufferers may believe that pollen allergy is more severe than HDM allergy-even though this is contradicted by literature data on clinical profiles and treatments [10, 15] and simply because pollen only allergic patients are free of symptoms between two seasons and know what being “normal” means. Even people with physician-diagnosed HDM allergy may consider that dust (rather than the HDMs in dust) is the trigger for their allergic symptoms [12].

Despite the availability of symptomatic medications (generally oral antihistamines and/or nasal corticosteroids) [8, 16, 17], HDM-allergic patients may not achieve adequate disease control. The concept of disease control goes beyond the mere observation of the frequency and intensity of allergic symptoms. Although there is no single definition of “disease control” in AR, an analogy with the guidelines on asthma control would prompt one to consider (i) disease exacerbations (e.g. the need for a previously unscheduled consultation with a physician, or the need to take rescue medication), (ii) the presence of impairments in leisure, sporting, professional and educational activities, (iii) objective measures of respiratory function [18]. In the survey described below, the participants reported non-optimal control at study entry on the basis of impaired activities and insufficient symptom relief.

Persistent, HDM-induced respiratory allergies are further complicated by the superposition of other (sometimes intermittent) allergies, such as those triggered by grass, tree and weed pollens to which they can be co-sensitized (and allergic). In a longitudinal, single-centre study in Sydney (Australia), Downie et al. characterized changes in the intensity of HDM-induced symptoms over a one-year period [19]. The typical domestic levels of HDM allergens in Australia are among the highest recorded worldwide; although two- to three-fold seasonal variations are seen in Sydney, the levels are always well over the presumed threshold for disease induction [14, 20, 21]. Downie et al. observed persistent nasal symptoms throughout the year, with pathologically high nasal symptom scores for 65 % of the 12-month study period [19]. Furthermore, increasing nasal symptom scores were predictive of the use of nasal medications. In pollen-co-sensitized patients, moderate seasonal variations in symptom scores had no effect on QoL and medication use [19]. The value of Downie et al.’s results are somewhat limited by the small number of participants (37 completed the study), the single-centre design and the high presumed domestic levels of HDM allergens.

Hence, the objective of the present observational, multicentre survey (performed throughout France, Italy and Spain) was to assess the self-reported disease profile of a large number of adult patients with a history of severe, poorly controlled, physician-diagnosed HDM respiratory allergy. We focused on poorly controlled patients because patients with mild-to-moderate, well-controlled HDM respiratory allergy (i) are less challenging to manage and (ii) may more readily display confounding symptoms related to allergens other than HDMs. Fortnightly telephone interviews were used to gather data on symptom prevalence and intensity, medical consultations, disease burden, QoL, and medication use over a 12-month real life period. In the first in a series of companion articles, we report on the survey’s methodology and the participants’ demographic characteristics, baseline disease profile and self-reported changes in symptom intensity and frequency over the 12-month survey period.

## Methods

### Survey design and ethical aspects

**Fig. 1:**
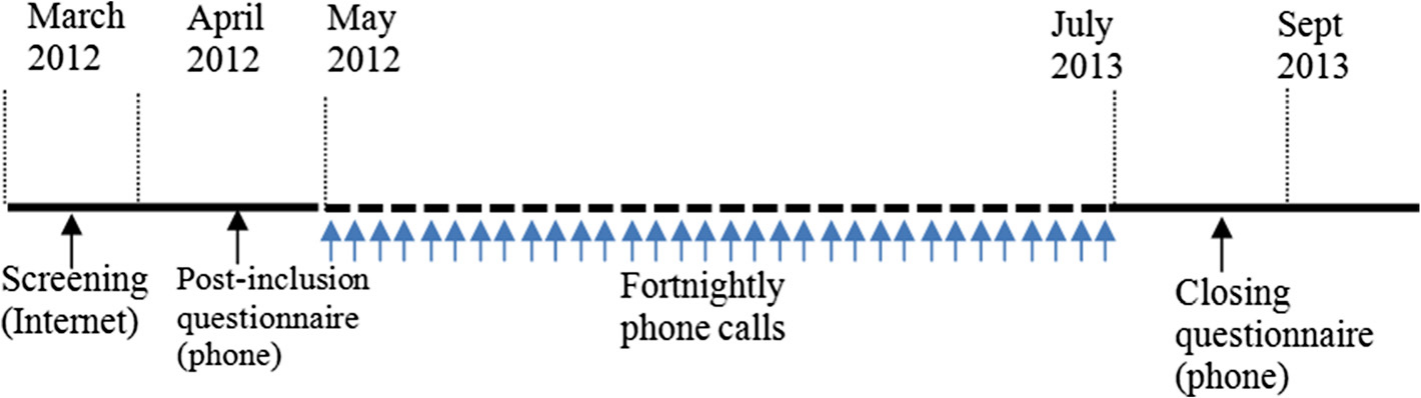
Study timeline

An observational, Internet- and telephone-based survey was performed in three Western European countries (Italy, France and Spain) between March 2012 and July 2013 (Fig. 1). In each country, members of nationwide patient panels (previously constituted by the study’s contract research organization (CRO) STETHOS (Sèvres, France)) were invited to participate in the present survey. In France, the study was registered with the French National Data Protection Commission (*Commission Nationale de l'Informatique et des Libertés*, CNIL). In Italy and Spain, we established that specific ethical and regulatory approval from independent ethics committees or health authorities was not required for a non-interventional, anonymous survey. In all three countries, the contract research organization complied with (i) the European Pharmaceutical Market Research Association’s guidelines, (ii) national legislation (notably with declarations to the national data protection committees and national medical associations) and (iii) an in-house code of good practice. Panel members were identified by a unique ID number (known to the CRO but not to the investigators). The anonymized format of the study database prevented the direct, nominative identification of panel members. The final data were analysed in a strictly de-identified, aggregated form and therefore could not be traced back to the respondent. The survey participants had provided their prior, general consent to participation in health-related surveys and subsequent exploitation of the anonymized data. Participants screened themselves for eligibility (i.e. through self-reporting) with a short Internet questionnaire (see below) and were not examined by a physician as part of the selection process. However, the participants all had to confirm that they had been diagnosed with HDM allergy by a physician.

### The screening questionnaire

In March and April 2012, potentially eligible people were screened with a 9-question Internet questionnaire (Additional file 1: Table S1). This questionnaire and the other survey questionnaires had been drafted in English, translated into local languages (Italian, French and Spanish) and then translated back into English for validation [22]. The inclusion criteria were as follows: age 18 or over; moderate to very severe symptoms of HDM allergy; at least three of the following symptoms: blocked nose, runny nose, itchy nose, difficulty breathing, cough, wheezing, sneezing, chest tightening, itchy eyes and tearing; physician-diagnosed HDM allergy (and potentially other allergies); a positive skin prick test or IgE assay for HDM allergens; more severe allergic symptoms in September, October, November or December; no previous or current AIT; use of at least one antihistamine or corticosteroid medication; symptoms not sufficiently controlled by current medication; a moderate to very strong impact of HDM allergy on QoL. In view of the descriptive nature of the study, a statistical calculation of the sample size was not performed and the number of participants was conditioned by financial and logistic parameters. The goal was to have approximately 100 participants per country (i.e. 315 in total) at the start of the 12-month assessment period and 80 per country at the end of the period). All questionnaire data were anonymous. No directly or indirectly nominative information was recorded. Included patients were modestly remunerated for their participation.

### The post-inclusion questionnaire

In April and May 2012, all included participants completed a 28-question post-inclusion questionnaire in an approximately 35-min telephone interview (Additional file 2: Table S2). The questionnaire was designed to probe the participant’s disease history and establish a baseline so as to compare at the end of the survey, the behaviour. The collected data included the first symptoms of HDM allergy, the types and frequencies of physician consultations, the time interval between the first symptoms and the first consultation, the presence of any other physician-diagnosed allergies, the types of symptoms experienced and their duration, frequency, seasonality and impact on health and life activities, the presence of any comorbidities, the use of allergen eviction measures, the types of allergy medications and their regimens, financial aspects (reimbursement, and costs linked to changes in the home or work environment) and, lastly, awareness and opinion of AIT. Again, all questionnaire data were anonymized. No directly or indirectly nominative information was recorded.

### The fortnightly status questionnaire

From late May 2012 to early July 2013, operators telephoned each participant every fortnight with a view to completing a 10-question telephone interview that lasted approximately 10 min (Additional file 3: Table S3). The questionnaire, which embedded the5 questions of the ARCT questionnaire, was used to collect data on the frequency and intensity of allergy-associated symptoms over the previous fortnight, the overall symptom severity, improvement, worsening or stability of symptoms, supposed reasons for any improvement or worsening, medication use, any consultations with physicians, the occurrence and treatment of comorbidities, and the HDM allergy’s impact on life activities and mood. A total of 28 fortnightly telephone interviews were scheduled. All questionnaire data were anonymized.

### The closing questionnaire

In August 2013, each participant was invited to complete a 24-question closing questionnaire during an approximately 20-min telephone interview (Additional file 4: Table S4). The closing questionnaire addresses the same topics as the post-inclusion questionnaire and was intended to identify trends in the management of the participant’s HDM allergy or any other major events (moving house, job changes, changes in the domestic and working environment, etc.) that may have influenced the disease signal and disease burden. All questionnaire data were anonymized.

### Data management

A descriptive analysis of the survey data was performed with SPSS software (version 15.0.1, IBM Corporation, Armonk, USA). Quantitative parameters are expressed as the mean, median and range, and qualitative parameters are expressed as the number and the percentage of the corresponding survey population or subpopulation.

## Results and discussion

### Screening and post-inclusion data

A total of 22,995 individuals completed the Internet screening questionnaire and 339 met all the inclusion criteria. Of these, 313 (*n* = 114 in Italy, 92 in France and 107 in Spain) were included in the study and completed the post-inclusion questionnaire. Even though the sample was not stratified for geographic location, the included participants were quite evenly distributed within each country. For example, 38 % of the 114 participants from Italy lived in the north of the country, 21 % lived in the middle regions and 41 % lived in the south. Similarly, participants in Spain were distributed between coastal locations (67 %) and inland locations (33 %). In France 59 % lived in the north of the country and 41 % in the south of the country. It was observed that participants wanted or needed to talk extensively about their disease. The post-inclusion questionnaire was meant to last for 35 min but often lasted longer.

The study population comprised a high proportion of women (67 % overall: 68 % for Italy, 72 % for France and 61 % for Spain; Table 1). This may have been due to self-selection bias because women are known to be more likely than men to participate in epidemiological surveys [23]. The mean age (Table 1) and age distribution highlighted the predominance of young adults in the study population: 18 to 24 years of age: 12 %; 25 to 34: 31 %; 35 to 44: 33 %, 45 to 54: 16 %; 55 to 64: 7 %; over 65: 1 %. This may reflect Internet use in the surveyed countries, with relatively fewer elderly people online [24]. Nevertheless, the age distribution appeared to be similar to that reported by Downie et al. in 2004; the latter researchers reported a mean (range) age of 33.4 (18–51) in their patients with HDM allergy only and 33.9 (18–64) in their patients with HDM allergy and pollen allergy [19].

**Table 1:** Characteristics of the survey population, according to the post-inclusion questionnaire

	Italy	France	Spain
Number of participants (*n* = M/F), % female.	114 (36/78), 68 %	92 (27/65), 72 %	107 (41/66), 61 %
Age: mean, median [range] (years):	37.5, 36 (18–63)	35.8, 36 (18–62)	38.2, 37 (18–68)
Time since first symptoms: mean, median [range] (years):	15.0, 16 (1–45)	17.8, 18 (1–51)	17.3, 13 (1–40)
Time interval between first symptoms and consultation with a specialist: mean, median [range] (months):	18.9, 4 (0.25–588)	28.4, 6 (0.25–360)	20.3, 11 (0.25–156)
Proportion of patients (%) having consulted the following physicians (mean number of visits per year):			
GP	92 % (3.4)	91 % (3.0)	70 % (3.9)
Allerist	87 % (2.0)	83 % (1.7)	70 % (1.4)
ENT specialist	27 % (2.3)	27 % (2.3)	15 % (1.7)
Dermatologist	24 % (1.6)	20 % (1.7)	8 % (2.6)
Pulmonologist	22 % (1.5)	32 % (1.6)	8 % (6.2)
Paediatrician	14 % (6.8)	9 % (1.0)	7 % (na)
Other	6 % (4.0)	2 % (1.5)	4 % (1.0)
Other self-reported allergies (% of patients):			
Grass pollen	79 %	67 %	67 %
Parietaria pollen	57 %	37 %	9 %
Cat dander	49 %	51 %	41 %
Dog dander	31 %	26 %	29 %
Olive pollen	27 %	41 %	35 %
Birch pollen	27 %	44 %	7 %
Moulds	24 %	29 %	35 %
Cypress pollen	21 %	48 %	9 %
None (i.e. HDMs only)	39 %	21 %	21 %
Proportion of patients (%) having consulted the following combinations of physicians:			
GP only	17 %	9 %	5 %
Allergist only	18 %	4 %	3 %
GP + allergist	33 %	25 %	31 %
GP + another specialist	7 %	7 %	5 %
GP + two specialists	11 %	29 %	26 %
GP + three or more specialists	4 %	22 %	28 %
Prevalence of co-morbidities (% of patients):			
Sinusitis	36 %	53 %	26 %
Otitis	13 %	18 %	9 %
Conjunctivitis	44 %	43 %	33 %
Headache	69 %	62 %	69 %
Time having used symptomatic medications (% of patients):			
Less than 2 years	7 %	3 %	3 %
2 to 5 years	35 %	28 %	21 %
6 to 10 years	22 %	39 %	30 %
11 to 20 years	21 %	23 %	27 %
More than 20 years	16 %	7 %	15 %
Degree of disease control (% of patients):			
Totally controlled	11 %	4 %	10 %
Well controlled	43 %	53 %	48 %
Moderately controlled	42 %	34 %	33 %
Poorly controlled	4 %	7 %	7 %
Not controlled at all	0 %	2 %	3 %
Proportion of patients (%) suffering from symptoms for more than 4 days in a week	70 %	58 %	54 %
Proportion of HDM-only allergic participants (%) with a peak in symptoms.	59 %	63 %	95 %
Proportion of patients (%) taking the following medications:			
Antihistamines	38 %	40 %	34 %
Nasal corticoids	7 %	12 %	10 %
Inhaled corticoids	3 %	3 %	9 %
Bronchodilators	7 %	12 %	15 %
Inhaled corticoids + bronchodilator	5 %	7 %	5 %
Oral or topical corticoids	12 %	3 %	4 %
Eye drops	4 %	6 %	4 %
Leukotriene receptor antagonists	2 %	4 %	3 %

As expected, the majority of participants (61 % in Italy, and 79 % in France and Spain) reported having another physician-diagnosed respiratory allergy (mainly due to grass pollen, followed by weed pollen, pet dander and tree pollen). In comparison, Downie et al. reported that (i) 57 % of their study participants were also sensitized to one or more pollens (ragweed, plantain, timothy grass or ryegrass), and (ii) some of the remaining 43 % were sensitized to allergens such as dander and moulds [19]. However, one cannot directly compare the prevalence of self-reported clinical allergy in the present study with the results of physician-administered sensitization tests and examinations (in Downie et al.’s study).

The median time since first symptoms of HDM allergy was over 13 years in all three countries. Most study participants had consulted within a year or two of disease onset; however, it implied that years of consultations and treatment offered had been inadequate and that patient’s needs were unmet and needed to be addressed. Participants were most likely to consult GPs (over 70 % of participants) and allergists (again over 70 % of participants). Fewer than 20 % of the participants had not consulted a specialist physician. The “GP+ allergist” combination was the most frequent (Table 1). The long disease duration and the multiple consultations are suggestive of “doctor-hopping” and unmet patient needs. In Spain, 28 % of the participants had consulted a GP and three different specialist physicians. Accordingly, over 93 % of the study participants had been taking symptomatic medication for more than 2 years (and for more than 20 years in some cases). The mean time since initiating symptomatic medications was 11.2 years in Italy, 10.1 years in France and 12.4 in Spain. When participants were asked which type of physician they consulted when allergic symptoms worsened, the most frequent response was “a GP” (according to 53 % of the participants in Italy, 51 % in France and 47 % in Spain). However, the second most frequent response (except in Italy) was “self-medication” with over-the-counter products (according to 22 % of the participants in France, 32 % in Spain and 4 % in Italy). Italian participants (36 %) were much more likely to consult an allergist (36 %, compared with 11 % in France and 12 % in Spain); this may reflect national differences in healthcare pathways and financial aspects.

The most common comorbidity was headache, although this was not necessarily related to the HDM allergy, although allergic rhinitis is a proven-risk factor for chronic rhinosinusitis [25–27]. The most common allergy-related comorbidity was conjunctivitis (in 33 to 44 % of the participants). There were some disparities in terms of disease control when comparing the results of the Internet-based screening questionnaire and those of the telephone-based post-inclusion questionnaire. As mentioned above, one of the inclusion criteria was the absence of total disease control. However, in response to the more detailed post-inclusion questionnaire (which notably included an opportunity to request clarifications on the meaning of the questions from the person conducting the telephone interview), between 4 and 11 % of the participants (depending on the country) reported total disease control and between 43 and 53 % reported good disease control (Table 1). Hence, only about half of the “poorly controlled” participants at selection truly had poor control in the post-inclusion questionnaire.

**Fig. 2:**
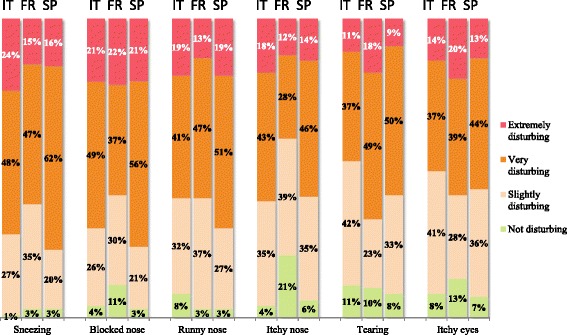
Impact of symptoms at baseline (according to the post-inclusion questionnaire)

There were few differences between symptoms (and indeed between countries) in terms of how bothersome nose and eye symptoms were judged to be at baseline (Fig. 2). Over 75 % of participants considered that their nasal symptoms were extremely or very bothersome. Chest and skin symptoms (wheezing, cough, chest pain, trouble breathing in general, trouble breathing when doing sport and eczema) were less bothersome, with between 14 and 52 % of the participants reporting them to be extremely or very bothersome.

**Fig. 3:**
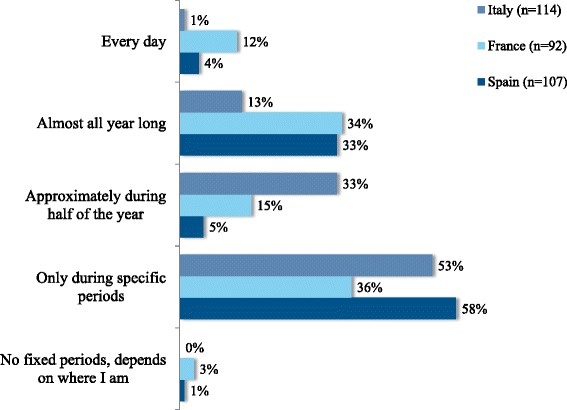
Frequency of symptoms at baseline (according to the post-inclusion questionnaire)

**Fig. 4:**
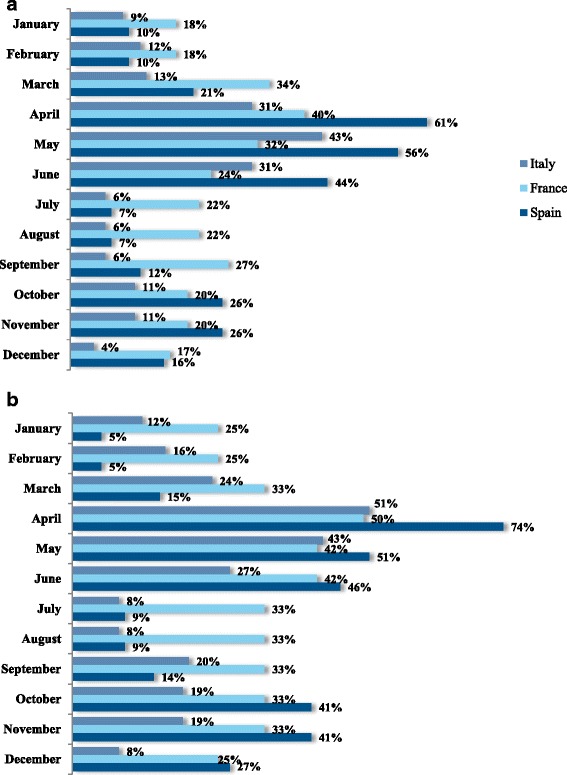
Self-reported history of seasonal variations in symptoms (according to the post-inclusion questionnaire) by the participants as a whole (top panel) and by participants allergic to HDMs only (bottom panel)

In terms of the recalled frequency of symptoms (i.e. prior to inclusion), most participants were only bothered during specific periods of the year (53 % in Italy, 36 % in France and 58 % in Spain. Nevertheless, a sizeable minority suffered from symptoms of HDM allergy every day or almost every day of the year (Fig. 3). When those only bothered during specific periods of the year were asked to specify the months of the year, spring and autumn peaks were apparent in all three countries (albeit to a lesser extent in France, where the HDM allergy appeared to be more persistent) (Fig. 4). Although these data may suffer from recall bias, they mirror the spring and autumn peaks typically observed in other studies of HDM allergy in the literature [28, 29]. Furthermore, these peaks were confirmed in participants with HDM allergy alone: a peak month of April was specified by 51, 50 and 74 % of intermittently affected participants in Italy, France and Spain, respectively. Overall, July and August were least frequently cited as seasonal peaks in symptoms by participants with HDM allergy alone, although there were marked differences between the three countries (8, 33 and 9 % for Italy, France and Spain in July, respectively). Only 5 % of Spanish participants stated that there were no specific periods in which symptoms were more bothersome (vs. 41 % in Italy and 37 % in France). The fact that the majority of “HDM only” participants recalled seasonal changes suggests that these peaks were not due to concomitant, intermittent allergies (e.g., pollen allergies) and that the peaks were similar irrespective of other associated sensitizations.

According to the post-inclusion questionnaire, antihistamines were the medications most frequently used to relieve allergic symptoms (Table 1), followed by bronchodilators, nasal corticosteroids and oral or topical corticosteroids. Most participants were taking more than one medication (80, 85 and 80 % in Italy, France and Spain, respectively). It is noteworthy that 11, 24 and 14 % of the participants in Italy, France and Spain were taking an antihistamine and at least three other medications. The great majority of French and Spanish participants were aware that AIT was a potential therapeutic option in HDM allergy (91 and 83 %, respectively). In contrast, only 39 % of the Italian participants were aware of AIT. However, a patient’s awareness of AIT did not mean that this treatment option was more likely to be suggested by a physician: in Spain, AIT had been suggested to 45 % of the AIT-aware participants. This figure was 54 % in France and 27 % in Italy.

### Changes over a year-long period, as reported by the fortnightly status interviews

The fortnightly status interviews’ completion rate averaged 75 % and ranged from 29 to 97 %, depending on the country and the period. The lowest completion rates were noted in August and September 2012 and June and July 2013. Overall, the response rates were highest in Spain and lowest in France.

**Fig. 5:**
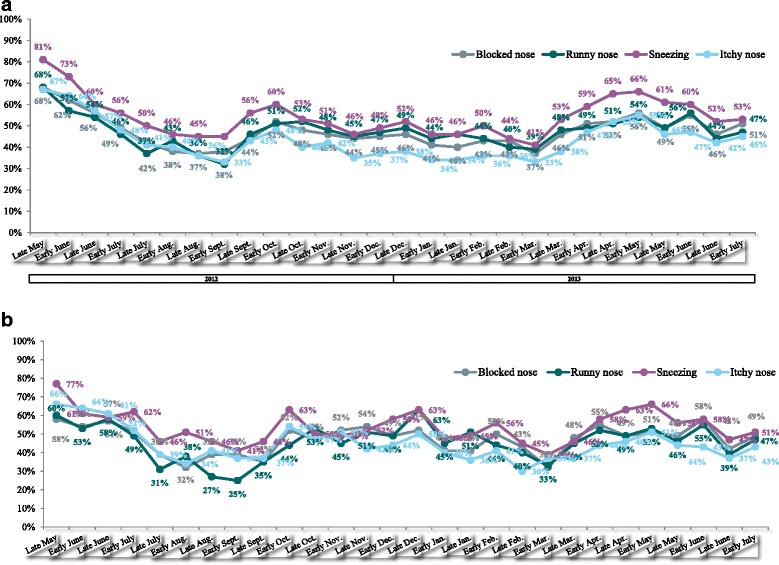
**a** Fortnightly status reports: the percentage of surveyed participants experiencing nasal symptoms (all patients, including those with concomitant allergies). **b** Fortnightly status reports: the percentage of surveyed participants experiencing nasal symptoms (“HDM only participants”, i.e. those with no concomitant allergies)

The fortnightly data on symptom prevalence confirmed the post-inclusion data. The time trends for the four nasal symptoms were similar; the fortnightly prevalence of blocked nose, runny nose, sneezing and itchy nose fell during the summer of 2012, rose in the autumn (peaking in early October), fell over the winter and rose again in the spring of 2013 (peaking in late May) (Fig. 5a). These trends were also confirmed by the reports from HDM-only participants, albeit with an additional (third) peak in late December (Fig. 5b) - suggesting that the spring and autumn peaks observed in the overall survey population were not due to (for example) tree/grass pollen in the spring and weed/tree pollen in the autumn. As mentioned above, Downie et al. noted moderate seasonal variations in symptom scores in “HDM + pollen-sensitive patients” but not in HDM-only patients [19]. Even in Downie et al.’s pollen-sensitive patients, the seasonal variations in symptom scores had no effect on QoL and medication use. Downie et al. suggested that this was due to fluctuating but persistently high allergen levels in the Sydney indoor environment.

**Fig. 6:**
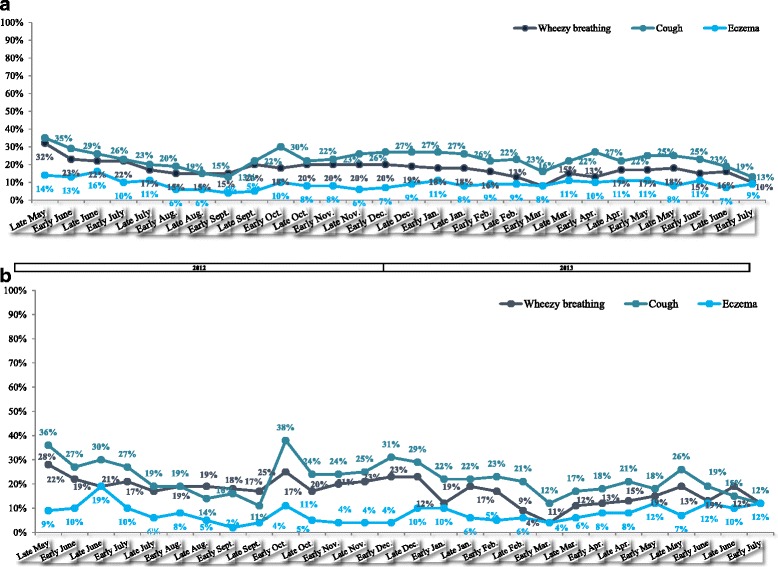
**a** Fortnightly status reports: the percentage of surveyed patients experiencing chest and eczema symptoms (all three countries pooled, all patients - including those with concomitant allergies). **b** the percentage of surveyed patients experiencing chest and eczema symptoms, according to fortnightly status reports during the survey period (all three countries pooled, patients with no concomitant allergies, i.e. HDM allergy only)

The fortnightly data revealed that chest and skin symptoms (Fig. 6) were generally less prevalent than nose and eye symptoms (Fig. 5). The prevalence of chest and skin symptoms also peaked in autumn and spring, albeit to a lesser extent than the nose and eye symptoms. Again, the results for the “HDM-only” participants were concordant with those for the survey population as a whole.

**Fig. 7:**
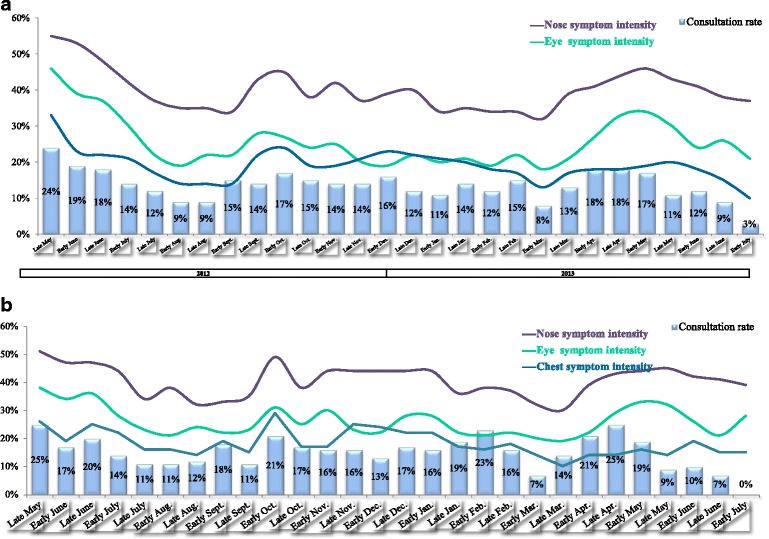
**a** symptom intensity and consultation with a physician, according to fortnightly status reports during the survey period (all three countries pooled and all patients, including those with concomitant allergies). **b** symptom intensity and consultation with a physician, according to fortnightly status reports during the survey period (all three countries pooled but only patients with no concomitant allergies, i.e. HDM allergy only)

The fortnightly data on the prevalence of allergic symptoms were confirmed by the participants’ judgment of whether their symptoms had improved, worsened or stayed the same over the previous fortnight (Additional file 5: Figure S1). The highest proportions of participants with worsened symptoms were noted between early September and late November 2012, and then again between late March and early May 2013. Very few participants reported that their symptoms had never improved (4 % overall; 2, 2 and 7 % in Italy, France and Spain, respectively) or never worsened (11 % overall; 12, 6 and 13 % in Italy, France and Spain, respectively) at any time during the study period. Again, the reports from HDM-only participants confirmed the overall picture, with the greatest proportion of “worsening” reported between early October and early December 2012 and then again between late March and early May 2013. A substantial proportion of participants (21 to 53 %, depending on the time of year) did not have an explanation (such as a change in medication use, a change in the environment or weather, greater exposure to dust, etc.) for the worsening or improvement in their symptoms, illustrating the complexity of the symptom profile in this context. Lastly, the autumn and spring peaks of symptom prevalence and worsening appeared to be associated with more frequent consultation of a physician (Fig. 7 and Additional file 6: Figure S2). In 2012, between 9 and 24 % of the participants had consulted a physician in the previous fortnight; unsurprisingly, the holiday month of August had the lowest consultation rates in 2012. In 2013, the peak in consultations (April and May) coincided with high rates of worsening.

In future research, it would be interesting to look for demographic and/or clinical risk factors associated with improvement or worsening. These fluctuating symptoms are likely to have had an impact on the participants’ degree of disease control, burden of disease (including financial factors and the need for medication), QoL (including sleep quality) and perception of disease. The fortnightly status interviews collected extensive data on these subjects, which will be described in a future companion article. By way of an example, the symptoms of HDM allergy markedly altered QoL between 2 and 4 days per fortnight on average, and led to time off work in up to 11 % of participants (although this proportion varied markedly over the 12-month study period)).

### Changes over the study period, as reported in the final questionnaire

A total of 214 participants (including 63 HDM-only participants) completed the final questionnaire (*n* = 72 in Italy, 70 in France and 72 in Spain). Compared with 12 months previously, 63 % of the respondents stated that their general condition was much the same (improvement: 32 %; worsening: 5 %). For HDM-only participants, these proportions were 73, 22 and 5 %. Both improvements and worsening were primarily attributed to changes in treatment and in the weather. 41 % of the respondents stated that the symptom of blocked nose was still very or extremely bothersome; this value was 36 % for sneezing, 34 % for a runny nose and 20 % for difficulty breathing. Strikingly, over 65 % of the respondents remembered their answers on this topic in the post-inclusion questionnaire completed 12 months previously, suggesting that their judgements of bothersome symptoms were reliable. When participants were asked to rate (on a 0 to 10 scale) the extent to which their symptoms were controlled by their medications, the mean score was 6.9 in Italy, 7.0 in France and 7.3 in Spain. Fewer than 6 % of participants reported completely uncontrolled disease. Use of three of more medications was not uncommon (28, 33 and 17 % of the respondents in Italy, France and Spain, respectively). Very few (*n* = 18, 8.4 %) participants had initiated AIT during the study (4, 1 and 13 in Italy, France and Spain, respectively).

The present study had several limitations, most of which apply to all self-reported, observational, Internet- or phone-based surveys. Fortnightly phone interviews may have induced a recall effect, and the closing questionnaire required participants to recall events and opinions up to 12 months previously. The data were self-reported over the Internet, which may have introduced bias. For practical reasons, self-reporting is frequent in “real-life” epidemiological surveys and (by definition) obligatory in online questionnaires that probe patient-reported outcomes (PROs) such as functional impairments and quality of life. However, self-reporting can introduce several different types of bias (for a review, see [30]). Firstly, subjective self-reporting is based on an individual’s subjective memories of his/her medical history and life events. The individual may therefore remember some events selectively, with unpleasant events and periods more likely to be remembered more clearly than pleasant ones. In addition, individual participant’s perception of risk can theoretically affect the validity of self-reported symptoms and perceived exposure is often an independent determinant of health outcome. Likewise, the recall period has an influence on responses; more recent events will be remembered more accurately than less recent ones. Secondly, social factors can influence recall and reporting; answers that are less socially acceptable or may be subject to stigma are less likely to be reported. However, it appears that the advantages of using PROs are currently outweighing the drawbacks, since the proportion of clinical trials at ClinicalTrials.gov reporting at least one PRO increased from 14 % prior to 2007 to 27 % between 2007 and 2013 [31].

This Internet-based survey was subject to selection bias. The present survey population was not representative of HDM-allergic people in general or of HDM-allergic patients consulting an allergist. For example, females were over-represented in the present survey (accounting for 67 % of the population) with regard to a typical patient population (e.g. 46.1 % in Trébuchon et al.’s study of French HDM-allergic patients consulting an allergist [8]). Furthermore, the present survey was subject to age bias. For example, data from the latest French national census (http://www.insee.fr/fr/themes/tableau.asp?reg_id=0&ref_id=NATnon02150) show that the 25 to 34, 35 to 44, 45 to 54 and 55 to 64 year age classes comprised respectively 12.1, 12.9, 13.5 and 12.5 % of the total population. In the present survey, these proportions were 31, 33, 16 and 7 %, respectively. Unsurprisingly, the Internet-based nature of our survey meant that young adults were markedly over-represented and older adults were underrepresented. This corresponds to coverage bias, since older adults are less likely to be Internet users [24]. The remuneration for study participation may also have introduced a socioeconomic status and age selection bias. Furthermore, the participants’ self-assessments of symptom severity and impact on QoL were not recorded with validated tools. The study questionnaires were custom tools and had not been extensively tested or psychometrically validated prior to the present study. In contrast to Downie et al.’s study, the clinical inclusion criteria were not verified by a physician, and interviews were not performed by physicians or other medical staff (although the majority of Downie et al.’s data also came from fortnightly phone interviews). Furthermore, we did not include a control group. In fact, Downie et al. monitored non-rhinitic subjects in order to establish the normal range for nasal symptom scores (defined as two standard deviations of the mean) [19]. Next, the absence of physician-validated clinical data meant that we did not perform any statistical analyses (notably comparisons of one country with another, and analyses of time trends). The fortnightly status interviews suffered from a variable completion rate, especially during holiday periods in the summer and at the end of the year. The study collected relatively little data on symptoms potentially associated with asthma. However, the fact that between 7 and 15 % of the participants had taken bronchodilators suggests that asthma was present. For cost reasons, HDM allergen levels were not measured in homes, although the value of this measurement is subject to debate [32–35]. Similarly, pollen counts across the many centres were not monitored and HDM avoidance measures were not uniform at baseline and differed from country to country and patient to patient. By comparison, Downie et al. was easily able to obtain pollen count data for Sydney [19].

Conversely, our study had some notable strengths: its international, multicentre design, the relatively large number of participants (at least when compared with the 37 in Downie et al.’s study [19]), the detailed post-inclusion and closing questionnaires, and the high frequency of follow-up interviews and detailed on participants with uncontrolled disease.

## Conclusions

Patients in France, Italy and Spain with severe HDM-induced allergic rhinitis and asthma experienced troublesome symptoms throughout the year, with peaks in spring, early autumn and (in HDM-only participants) late winter. A substantial proportion of participants did not have an explanation for the worsening or improvement in their symptoms-illustrating the complexity, variability and the hidden aspect of the disease in this context. At the end of the survey period, nasal symptoms were stated to be very or extremely bothersome by over 40 % of respondents, suggesting the persistence of unmet needs.

## Abbreviations

AIT, allergy immunotherapy; AR, allergic rhinitis; HDM, house dust mite; IgE, immunoglobulin E; QoL, quality of life.

## Additional files

Additional file 1: Table S1. Screener questionnaire. (DOCX 27 kb) Additional file 2: Table S2. Post inclusion Questionnaire. (DOCX 33 kb) Additional file 3: Table S3. Routine Questionnaire. (DOCX 33 kb) Additional file 4: Table S4. Final Questionnaire. (DOCX 25 kb) Additional file 5: Figure S1. Whole survey population - late May to December 2012/Whole survey population - January to July 2013. (JPG 413 kb) Additional file 6: Figure S2. Late May to December 2012/January to July 2013. (JPG 206 kb)
