# Supplementary material for: “A year-long, fortnightly, observational survey in three European countries of patients with respiratory allergies induced by house dust mites: Methodology, demographics and clinical characteristics”
Source: BMC Pulm Med. 2016 May 23;16:85. doi: 10.1186/s12890-016-0246-9 (PMC4877752; doi:10.1186/s12890-016-0246-9)
Supplement: Additional file 1: Table S1. — Screener questionnaire. (DOCX 27 kb) [file 12890_2016_246_MOESM1_ESM.docx]

**SCREENER QUESTIONNAIRE**

**TO ENSURE THE RECRUITMENT OF THE RIGHT TARGET POPULATION**

1. ***Do you suffer from allergic rhinitis due to house dust mites allergy?***

🞏 Yes 🞏 no

***If no 🡪 exit***

1. ***During which months do you generally suffer most from your house dust mites allergy ?***

🞏 January 🞏 February 🞏 March 🞏 April 🞏 May 🞏 June
🞏 July 🞏 August 🞏 September 🞏 October 🞏 November 🞏 December

🞏 all year long

***If nothing between September and December (or all year long)🡪 exit***

1. ***Have you consulted a specialist doctor for your house dust mites allergy?***

🞏Yes: please give the speciality or specialities 🞏no

***If no 🡪 exit***

1. ***Did this specialist doctor confirm the diagnosis of house dust mites allergy?***

🞏Yes : how: 🞏Prick tests (skin tests) 🞏IgE blood tests 🞏Both

🞏 No

***If no 🡪 exit***

1. ***Are you following or have you ever followed a desensitisation treatment (specific Immunotherapy)?***

🞏Yes 🞏no

***If yes 🡪 exit***

***Q6/ Do your house dust mites allergy provoke one or various of the following allergic symptoms?
(several answers possible)***🞏 Nasal congestion (blocked nose) 🞏 Breathing difficulties 🞏Nasal discharge (runny nose)
🞏Cough 🞏 Wheezing 🞏 Sneezing

🞏chest tightness 🞏 Nasal itching 🞏 Eye itching 🞏 Watery eyes ***If less than 3 different symptoms 🡪 exit***

***Q7/ How would you rank the intensity of your allergic symptoms to house dust mites?***

🞏Mild 🞏moderate 🞏severe 🞏 very severe

***If mild 🡪 exit***

***Q8/ How would you grade the impact of your house dust mites allergy on your quality of life (daily professional/personal activities):***

🞏 None 🞏 Low 🞏 Moderate 🞏 Important 🞏 Very high

***If none or low 🡪 exit***

***Q9/ What types of treatment do you take?***

🞏 Anti-histamines (tablets or eye drops) 🞏 Nasal corticosteroids (nose spray)

🞏 Inhaled corticosteroids (inhaler) 🞏 Anti-leukotrienes 🞏 Decongestants

please give the names of all of the medication (nb: we add this question to be sure of the medication taken: patients are not often able to classify the medication into categories!):

***If none 🡪 exit***

***Q10/ Do you feel that your symptoms are sufficiently controlled by these treatments (ie controlled = symptoms disappear)?***

☐Not controlled at all ☐ Very slightly controlled ☐ Somewhat controlled ☐ Well controlled ☐Completely controlled

***If well or completely controlled 🡪 exit***

PATIENT PROFILE

Age: /___/___/

Gender: 🞏Male 🞏 Female
