# Supplementary material for: “A year-long, fortnightly, observational survey in three European countries of patients with respiratory allergies induced by house dust mites: Methodology, demographics and clinical characteristics”
Source: BMC Pulm Med. 2016 May 23;16:85. doi: 10.1186/s12890-016-0246-9 (PMC4877752; doi:10.1186/s12890-016-0246-9)
Supplement: Additional file 2: Table S2. — Post inclusion Questionnaire. (DOCX 33 kb) [file 12890_2016_246_MOESM2_ESM.docx]

**POST INCLUSION QUESTIONNAIRE**

**1^st^ questionnaire asked to patients once they are recruited (25 minutes length)**

PATIENT PROFILE

Age: /___/___/

Gender: 🞏Male 🞏 Female

**1st part: Background and impact of the "ALLERGY TO HOUSE DUST MITES "**

1. ***For how long have you been suffering from your allergy to house dust mites?***

/_____/ years **or** /_____/ months

1. ***Which of the following doctor(s) have you consulted for your allergy, since suffering from it?***

🞏GP 🞏Allergist 🞏ENT 🞏Dermatologist

🞏Chest Specialist 🞏Paediatrician 🞏Other: specify

1. ***Since suffering from this allergy, how often have you visited these doctors for your allergy?***

- GP consultations: /___/___/ times/year for the last /___/___/ years

- Allergist consultations /___/___/ times/year for the last /___/___/ years

- ENT consultations /___/___/ times/year for the last /___/___/ years

- Chest Specialist consultations /___/___/ times/year for the last /___/___/ years

- Dermatologist consultations /___/___/ times/year for the last /___/___/ years

- Paediatrician consultations /___/___/ times/year for the last /___/___/ years

- Other 🡪 Specify: _____ /___/___/ times/year for the last /___/___/ years

1. ***How long did you wait between the first symptoms of your House dust mites allergy and your first consultation with a specialist doctor for these symptoms?***

/_____/ years **or** /_____/ months **or** /_____/ weeks
**or** 🞏 Don’t know

1. ***Have you also been diagnosed as suffering from any other respiratory allergy?***

🞏 No I am only allergic to house dust mites

🞏 Yes I am also allergic to:

🞏Cat 🞏Dog 🞏Grass pollen 🞏Birch pollen 🞏Ash pollen 🞏Olive pollen

🞏Cypress pollen 🞏Parietaria pollen 🞏Ragweed pollen 🞏Moulds 🞏Other please specify:

1. ***Today, in the case of an exacerbation of your allergic symptoms to house dust mites, who do you consult?***

🞏GP 🞏Allergist 🞏ENT
🞏Dermatologist 🞏Chest specialist 🞏Paediatrician 🞏Other: specify

🞏I don’t go to the doctor, I go to the chemist/pharmacist

🞏I don’t go to the doctor, I use self-medication

1. ***Regarding your house dust mite allergy, please describe to which extent you are bothered by the following symptoms***

| ***Symptoms*** | ***Not bothered at all*** | ***Slightly bothered*** | ***Very bothered*** | ***Extremely bothered*** |
| --- | --- | --- | --- | --- |
| ***Sneezing*** | 🞏 | 🞏 | 🞏 | 🞏 |
| ***Blocked nose*** | 🞏 | 🞏 | 🞏 | 🞏 |
| ***Runny nose*** | 🞏 | 🞏 | 🞏 | 🞏 |
| ***Itchy nose*** | 🞏 | 🞏 | 🞏 | 🞏 |
| ***Watery eyes*** | 🞏 | 🞏 | 🞏 | 🞏 |
| ***Itchy eyes*** | 🞏 | 🞏 | 🞏 | 🞏 |
| ***Wheezing*** | 🞏 | 🞏 | 🞏 | 🞏 |
| ***Cough*** | 🞏 | 🞏 | 🞏 | 🞏 |
| ***Chest tightness*** | 🞏 | 🞏 | 🞏 | 🞏 |
| ***Breathing difficulties***  ***Is it only when doing sports or exercise?*** | 🞏  🞏 | 🞏  🞏 | 🞏  🞏 | 🞏  🞏 |
| ***Eczema*** | 🞏 | 🞏 | 🞏 | 🞏 |
| ***Other, please specify:*** | 🞏 | 🞏 | 🞏 | 🞏 |
| ***Other, please specify:*** | 🞏 | 🞏 | 🞏 | 🞏 |

1. ***On the overall, are you bothered by these symptoms***

🞏Every day (but with more or less strong symptoms)

🞏Almost throughout the year (there are very few days when I didn’t have any symptoms)

🞏More or less half the year, on average between ---- and ----- (state months)

🞏 Only on very specific period (s) in the year: (please state the months) on average

🞏Other, please specify:

1. ***And more precisely, in a period of symptoms, would you say that you suffer from these symptoms:***

🞏Less than 4 days a week

🞏More than 4 days a week

🞏Less than 4 consecutive weeks

🞏More than 4 consecutive weeks

1. ***Is there a specific period in the year with a peak in your symptoms***

🞏No

🞏Yes: please specify:

1. ***Apart from these symptoms do you frequently suffer from the following conditions***

🞏Sinusitis /___/ times/year on average

🞏Otitis /___/ times/year on average

🞏Conjunctivitis /___/ times/year on average

🞏Headache /___/ times/year on average

🞏 Dental mal occlusion /___/ times/year on average

🞏 Low energy /___/ times/year on average

🞏 Depression /___/ times/year on average

🞏Other:

1. ***Does your House dust mites allergy has an impact on :***

|  | ***No impact*** | ***Slight impact*** | ***Some impact*** | ***Important impact*** | ***Very important impact*** |
| --- | --- | --- | --- | --- | --- |
| Your day-to-day activities (housework, leisure activities, DIY, sports, gardening…) | 🞏 | 🞏 | 🞏 | 🞏 | 🞏 |
| Your professional activity | 🞏 | 🞏 | 🞏 | 🞏 | 🞏 |
| Your relations with others / social and personal activities (family, friends, colleagues) | 🞏 | 🞏 | 🞏 | 🞏 | 🞏 |
| Your sleep (difficulties to fall asleep or nocturnal awakenings) | 🞏 | 🞏 | 🞏 | 🞏 | 🞏 |
| Your irritability | 🞏 | 🞏 | 🞏 | 🞏 | 🞏 |
| Feeling tired/ high fatigue | 🞏 | 🞏 | 🞏 | 🞏 | 🞏 |
| Your quality of life | 🞏 | 🞏 | 🞏 | 🞏 | 🞏 |

1. ***Please indicate 3 examples which give the best demonstration of the impact of your House dust mites allergy on your quality of life:***

***1.***

***2.***

***3.***

1. ***Are there some activities that you were used to do and had to quit linked to your allergy (work, sports, social activities….)?***
2. ***How would you describe, in 3 lines, your frame of mind regarding your House dust mites allergy: how do you live with it? Do you think you will get rid of it one day?***

(your allergic symptoms/ the impact on your quality of life / the treatments available / the medical consultations)

**2nd part: Current avoidance measures and Treatments**

1. ***Since you know you are allergic to mites, have you applied some specific avoidance measures?***

🞏anti mite mattress cover

🞏anti mite spray (acaricide)

🞏bedding wash every */___/* *(please state the frequency)*

🞏house cleaning every */___/* *(please state the frequency)*

🞏special vacuum cleaner

🞏arrangement of the house (take out the carpets/ soft toys/stuffed animals/sofa/curtains )

🞏none

🞏other : please state the different measures you have taken :

1. ***Have you also applied some specific measures to your workplace?***

🞏 No

🞏 Yes, please specify:

1. ***On average, to what amount would you estimate your personal expenses for house fitting to avoid any mite allergy crisis at home?***

About /______/ € in total

1. ***In relation to your House dust mites allergy, please describe the treatment(s) you take?*** *Please give* ***all*** *the treatments (names of medication) that you take when you have House dust mites allergy symptoms?*

| ***MEDICATION*** | | ***TREATMENT DURATION*** | | ***TREATMENT OBTENTION*** | | |
| --- | --- | --- | --- | --- | --- | --- |
| name of medication | ***Number of doses per day*** |  | ***Total duration of the treatment***  *(in days or months)* | ***Prescribed by a doctor*** | ***Self medication*** | ***Recommended by the chemist/pharmacist*** |
| **1.** | /_______/ Doses /______/ times per day | 🞏Taken as a cure  🞏Taken on-demand (only in case of symptoms) | /_______/ days or /______/ months  (Which particular months: ) | 🞏GP  🞏Specialist: specify: | 🞏 | 🞏 |
| **2.** | /_______/ Doses /______/ times per day | 🞏Taken as a cure  🞏Taken on-demand (only in case of symptoms) | /_______/ days or /______/ months  (Which particular months: ) | 🞏GP  🞏Specialist: specify: | 🞏 | 🞏 |
| **3.** | /_______/ Doses /______/ times per day | 🞏Taken as a cure  🞏Taken on-demand (only in case of symptoms) | /_______/ days or /______/ months  (Which particular months: ) | 🞏GP  🞏Specialist: specify: | 🞏 | 🞏 |
| **4.** | /_______/ Doses /______/ times per day | 🞏Taken as a cure  🞏Taken on-demand (only in case of symptoms) | /_______/ days or /______/ months  (Which particular months: ) | 🞏GP  🞏Specialist: specify: | 🞏 | 🞏 |
| **5.** | /_______/ Doses /______/ times per day | 🞏Taken as a cure  🞏Taken on-demand (only in case of symptoms) | /_______/ days or /______/ months  (Which particular months: ) | 🞏GP  🞏Specialist: specify: | 🞏 | 🞏 |

1. ***During your last period of symptoms, have you needed to take an additional treatment not prescribed by your doctor to treat your allergic rhinitis?***

☐4 nights or more per week ☐2 to 3 nights per week ☐1 night per week ☐1 to 2 times in all ☐Never

1. ***To what amount would you estimate your personal expenses for medication to be per year (taking out the eventual reimbursement from healthcare system and private insurance)?***

About /______/ € /month meaning /______/ € /year

1. ***For how many years have you been taking these treatments to relieve your House dust mites allergy symptoms?***

/______/ years - /______/ months

1. ***Do you feel that your symptoms are sufficiently controlled by these treatments (i.e. controlled = symptoms disappear)?***

☐Not controlled at all ☐ Very slightly controlled ☐ Somewhat controlled

☐ Well controlled ☐Completely controlled

***Please grade it on a scale from 0 (not controlled) to 10 (completely controlled):***

1. ***Have you ever heard about the desensitisation treatments?***

🞏 yes **⇨ Q23** 🞏 no  **(STOP)**

1. **If Q22 = YES*: Have you already been offered a desensitisation treatment for your House dust mites allergy?***

🞏 YES **⇨ Q 24** 🞏 NO  **(STOP)**

1. **If Q23 = YES*: Which doctor proposed this treatment to you?***

🞏 gp 🞏 allergist 🞏 ent 🞏 dermatologist

🞏 chest specialist 🞏 paediatrician 🞏 Other, please specify: _______

1. ***Why did you refuse this/these desensitisation treatment(s)?*** *(please give details here of all of your reasons for refusal, for each treatment refused)*
