# Supplementary material for: “A year-long, fortnightly, observational survey in three European countries of patients with respiratory allergies induced by house dust mites: Methodology, demographics and clinical characteristics”
Source: BMC Pulm Med. 2016 May 23;16:85. doi: 10.1186/s12890-016-0246-9 (PMC4877752; doi:10.1186/s12890-016-0246-9)
Supplement: Additional file 3: Table S3. — Routine Questionnaire. (DOCX 33 kb) [file 12890_2016_246_MOESM3_ESM.docx]

**ROUTINE QUESTIONNAIRE**

**ADMINISTERED TO RECRUIED PATIENTS EVERY 2 WEEKS BY PHONE OVER ONE YEAR** *(DISCONTINUATION IN SUMMER)*

***Q1 / On the last 2 weeks, have you experienced the following symptoms related to your dust mite allergy? (check all that apply)***

**☐** sneezing If yes: how many days….: ___ Peak: Grade from 0 (no symptom) to 10 (very severe symptoms)
☐ blocked nose If yes: how many days….: ___ Peak: Grade from 0 (no symptom) to 10 (very severe symptoms)

☐ runny nose If yes: how many days….: ___ Peak: Grade from 0 (no symptom) to 10 (very severe symptoms)

☐ Itchy nose If yes: how many days….: ___ Peak: Grade from 0 (no symptom) to 10 (very severe symptoms)

☐ watery eyes If yes: how many days….: ___ Peak: Grade from 0 (no symptom) to 10 (very severe symptoms)

☐ itchy eyes If yes: how many days….: ___ Peak: Grade from 0 (no symptom) to 10 (very severe symptoms)

☐ wheezing If yes: how many days….: ___ Peak: Grade from 0 (no symptom) to 10 (very severe symptoms)

☐ cough If yes: how many days….: ___ Peak: Grade from 0 (no symptom) to 10 (very severe symptoms)

☐ eczema If yes: how many days….: ___ Peak: Grade from 0 (no symptom) to 10 (very severe symptoms)
☐ Other, specify:.. If yes: how many days….: ___ Peak: Grade from 0 (no symptom) to 10 (very severe symptoms)

***Q2 / Overall, how would you rate your level of allergic symptoms on these last 2 weeks***

☐ Mild ☐ Moderate ☐ Severe

***Please grade on a scale from 0 (mild) to 10 (severe) :***

***Q3/ Overall, have your symptoms improve/ deteriorate since the previous 2 weeks?***

☐ Improve ☐ Deteriorate

***Q3 / Over the same period, what drugs did you take to treat your allergy to house dust mites?***

***(List of all available treatment in the country will be given to the interviewer)***

***AERIUS  ALERDUAL  ALLERGEFON  ALLERGOCOMOD  ALLERGODIL  ALLERGODOSE  ALLOPTREX  ALMIDE  Antihistaminiques autre APAISYL APHILAN  ATARAX 
BECONASE BECOTIDE CELESTAMINE  CETIRIZINE  CLARINASE REPETABS  CLARITYNE  Corticoïdes voie nasale autre Corticoïdes voie orale autre CROMABAK  CROMADOSES  CROMEDIL  CROMOGLICATE  CROMOPTIC  CROMOSOFT  DERINOX  DETURGYLONE  DEXAMETHASONE  DIMEGAN  EMADINE  FLIXONASE FLIXOTIDE FORADIL GRANIONS MANGANESE  HISMANAL HUMEX RHINITE ALLERGIQUE  HYPOSTAMINE  INTERCRON  ISTAMYL  KESTIN  LEVOPHTA  LODOXAL  LOMUSOL  MEREPRINE  MISTALLINE  MIZOLLEN  MULTICROM  NAABAK  NAAXIA  NALCRON  NASACORT NASALIDE NASONEX  OPATANOL  OPHTACALM  OPTICRON  PERIACTINE  PIVALONE POLARAMINE  POLYDEXA PRIMALAN  PULMICORT PURIVIST  QUITADRILL  REACTINE  RHINAAXIA  RHINIREX  RHINOCORT 
SERETIDE SEREVENT SINGULAIR SOLUCORT SYMBICORT TELFAST  TILAVIST  TINSET  VIRLIX  XYZALL  ZADITEN ZYRTEC  ZYRTEC SET
FOR ANY MEDICATION specify the number of days with drug taking and if the drug was obtained on prescription or OTC?***

***+ question again "Are you sure you did not take any other treatment?”.***

***Q5 / Have you seen a doctor for your allergy to dust mites in the last 15 days?***
☐ No consultation
☐ GP: how often:
☐ Specialist:
 ☐ Allergist ☐ Dermatologist ☐ ENT

☐ Pulmonologist ☐ Pediatrician ☐ Other: specify

***Q6 / During the same time have you had the following diseases?***

| ☐ Sinusitis | If yes: Treatment taken: |
| --- | --- |
| ☐ Otitis | If yes: Treatment taken: |
| ☐ Asthma | If yes: Treatment taken: |
| ☐ Headache | If yes: Treatment taken: |
| ☐ Conjunctivitis | If yes: Treatment taken: |
| ☐Low energy | If yes: Treatment taken: |
| ☐Depression | If yes: Treatment taken: |
| ☐ Other; specify: | If yes: Treatment taken: |

***Q7 / During the last 15 days, has your allergy to dust mites affected you in each of the following:***

| ***During the last 2 weeks, has your allergic rhinitis had an effect on your professional / scholar activities? ^(1)^***  ☐Permanently ☐Very often ☐Often ☐Not often ☐Never |
| --- |
| ***During the last 2 weeks, has your allergic rhinitis made you irritable? ^(1)^***  ☐Permanently ☐Very often ☐Often ☐Not often ☐Never |
| ***During the last 2 weeks, has your allergic rhinitis disturbed your sleep (going to sleep/ waking at night)? ^(1)^***  ☐Permanently ☐Very often ☐Often ☐Not often ☐Never |
| ***During the last 2 weeks, have you needed to use an additional treatment not prescribed by your doctor to treat your allergic rhinitis? ^(1)^***  ☐4 nights or more/week ☐ 2 to 3 nights/week ☐1 night/week ☐ 1 to 2 times in all ☐ Never  ***If so, how much have you paid for it? ……………..€*** |
| ***During the last 2 weeks, how would you assess your allergic rhinitis? ^(1)^***  ☐Not controlled at all ☐ Very slightly controlled ☐ Somewhat controlled ☐ Well controlled ☐Completely controlled  Please grade on a scale from 0 (not controlled) to 10 (completely controlled): |
| - ***During the last 2 weeks, has your allergic rhinitis made you tired during the day?*** - ☐Permanently ☐Very often ☐Often ☐Not often ☐Never |
| - ***During the last 2 weeks, has your allergic rhinitis had an effect on your social/personal activities (going out with friends, visiting family…)?*** - ☐Permanently ☐Very often ☐Often ☐Not often ☐Never |
| - ***During the last 2 weeks, have you felt ill at ease in public due to your allergic rhinitis symptoms (repetitive sneezing, continuous cough, runny nose, swollen eyes)?*** - ☐Permanently ☐Very often ☐Often ☐Not often ☐Never |
| - ***Number of days with a real alteration of Quality of life in the period:*** - ***Number of missed working/university/school days (linked to your mites allergy):*** |

***Q8 / During the last 15 days overall how would you rate your level of discomfort caused by your allergy to dust mites?*** ☐Permanently ☐Very often ☐Often ☐Not often ☐Never

***Q9 / During the last 15 days, how did you feel about your allergy to dust mites?*** ☐ I am a lot annoyed ☐ I am annoyed ☐ I somewhat bear it ☐ I bear it ☐ I live with it very well ***Q10 / In our last interview you said*** *“grade last interview,"* ***how do you explain the improvement / deterioration of your answer?***

☐I have taken another medicine

☐I have increased the dose or frequency of intake of my medicine

☐I have decreased the dose or frequency of intake of my medicine

☐I have changed things in my environment : *please specify:*

☐I have been in contact with other allergens to which I am allergic

☐Other, please specify:____________________________________

☐I don’t know
