# Supplementary material for: “A year-long, fortnightly, observational survey in three European countries of patients with respiratory allergies induced by house dust mites: Methodology, demographics and clinical characteristics”
Source: BMC Pulm Med. 2016 May 23;16:85. doi: 10.1186/s12890-016-0246-9 (PMC4877752; doi:10.1186/s12890-016-0246-9)
Supplement: Additional file 4: Table S4. — Final Questionnaire. (DOCX 25 kb) [file 12890_2016_246_MOESM4_ESM.docx]

**FINAL QUESTIONNAIRE**

**Last questionnaire addressed to patients at the end of study (Duration: 20 minutes)**

1. ***You’ve taken part to a one year long study. After those 12 months would you say that your general condition concerning your house dust mite allergy?***

🞏 Improved

🞏 Worsened

🞏 Remained the same

1. ***Why?***

/__________________________________________________________________________________________________________________________________________________________________________/

1. ***In relation to your house dust mite allergy, please describe to what degree the following symptoms bothered you over the last 12 months:***

- **Column C**
- **Repeat in column A Post Inclusion Questionnaire answer**

1. ***Do you remember how you answered that same question one year ago for each symptom?***

- **Column B**

1. ***For each symptom: mention to the patient the actual answer he / she gave 1 year ago (if different from the one remembered). If ‘Remembered answer’ (column B) / ‘Post inclusion actual answer’ (column A) is different from ‘Final questionnaire answer’ (column C): how would you explain the change in your answer vs. last year?***

/__________________________________________________________________________________________________________________________________________________________________________/

| **Table for questions Q3, Q4 & Q5** | | | | | | | | | | | | |
| --- | --- | --- | --- | --- | --- | --- | --- | --- | --- | --- | --- | --- |
| ***Symptoms*** | ***Not bothersome*** | | | ***Slightly bothersome*** | | | ***Very bothersome*** | | | ***Extremely bothersome*** | | |
|  | **A** | **B** | **C** | **A** | **B** | **C** | **A** | **B** | **C** | **A** | **B** | **C** |
|  | *Post inclusion actual answer* | *Post inclusion remem-bered answer* | *Final question-naire answer (Q4)* | *Post inclusion actual answer* | *Post inclusion remem-bered answer* | *Final question-naire answer (Q4)* | *Post inclusion actual answer* | *Post inclusion remem-bered answer* | *Final question-naire answer (Q4)* | *Post inclusion actual answer* | *Post inclusion remem-bered answer* | *Final question-naire answer (Q4)* |
| Sneezing | 🞏 | 🞏 | 🞏 | 🞏 | 🞏 | 🞏 | 🞏 | 🞏 | 🞏 | 🞏 | 🞏 | 🞏 |
| Blocked nose | 🞏 | 🞏 | 🞏 | 🞏 | 🞏 | 🞏 | 🞏 | 🞏 | 🞏 | 🞏 | 🞏 | 🞏 |
| Runny nose | 🞏 | 🞏 | 🞏 | 🞏 | 🞏 | 🞏 | 🞏 | 🞏 | 🞏 | 🞏 | 🞏 | 🞏 |
| Itchy nose | 🞏 | 🞏 | 🞏 | 🞏 | 🞏 | 🞏 | 🞏 | 🞏 | 🞏 | 🞏 | 🞏 | 🞏 |
| Tearing | 🞏 | 🞏 | 🞏 | 🞏 | 🞏 | 🞏 | 🞏 | 🞏 | 🞏 | 🞏 | 🞏 | 🞏 |
| Itchy eyes | 🞏 | 🞏 | 🞏 | 🞏 | 🞏 | 🞏 | 🞏 | 🞏 | 🞏 | 🞏 | 🞏 | 🞏 |
| Wheezing | 🞏 | 🞏 | 🞏 | 🞏 | 🞏 | 🞏 | 🞏 | 🞏 | 🞏 | 🞏 | 🞏 | 🞏 |
| Cough | 🞏 | 🞏 | 🞏 | 🞏 | 🞏 | 🞏 | 🞏 | 🞏 | 🞏 | 🞏 | 🞏 | 🞏 |
| Chest tightening | 🞏 | 🞏 | 🞏 | 🞏 | 🞏 | 🞏 | 🞏 | 🞏 | 🞏 | 🞏 | 🞏 | 🞏 |
| Difficulty breathing | 🞏 | 🞏 | 🞏 | 🞏 | 🞏 | 🞏 | 🞏 | 🞏 | 🞏 | 🞏 | 🞏 | 🞏 |
| Difficulty breathing specifically when you participate in sports or exercise | 🞏 | 🞏 | 🞏 | 🞏 | 🞏 | 🞏 | 🞏 | 🞏 | 🞏 | 🞏 | 🞏 | 🞏 |
| Eczema | 🞏 | 🞏 | 🞏 | 🞏 | 🞏 | 🞏 | 🞏 | 🞏 | 🞏 | 🞏 | 🞏 | 🞏 |

1. ***In comparison with 1 year ago, would you say that the symptoms induced by your house dust mites allergy are:***

🞏 More bothering today than 1 year ago

🞏 Less bothering today than 1 year ago

🞏 As bothering today as they were 1 year ago

1. ***If Q6=Less bothering today than 1 year ago: how would you explain that improvement?***

 Took a treatment

 Took a different treatment

 Increased treatment dose / frequency

 Changed something in environment at home, please specify: /____________________/

 Changed something in environment at work, please specify: /____________________/

 Been less in contact with other allergens

 Better weather

 I have stopped smoking

 Other, please specify: /____________________/

 Don’t know

1. ***If Q6=More bothering today than 1 year ago: how would you explain that worsening?***

 Stopped taking my treatment

 Took a different treatment

 Decreased treatment dose / frequency

 Changed something in environment at home, please specify: /____________________/

 Changed something in environment at work, please specify: /____________________/

 Been more in contact with dust

 Been more in contact with other allergens

 Worst weather

 I have started smoking

 Other, please specify: /____________________/

 Don’t know

1. ***Has anything changed in your personal life during the last 12 months? (did you move, have your house refurbish, have a pet, etc.)***

☐ Yes ☐ No
If Yes: please explain /________________________________/

1. ***Has anything changed in your professional life during the last 12 months? (did you change job, change office, have your office refurbish, etc.)***

☐ Yes ☐ No
If Yes: please explain /________________________________/

1. ***Which physician would you say has been your ‘physician of reference’ for your house dust mite allergy over the last 12 months?***

🞏 General Practitioner

🞏 Allergist

🞏 ENT

🞏 Dermatologist

🞏 Pulmonologist

🞏 Paediatrician

🞏 Other, please specify: /________________________________/

1. ***What would you say has been your ‘treatment scheme of reference’ for your house dust mite allergy over the last 12 months? (you may have taken other drugs during the year, we’re interested here in the drug(s) you took more often, as ‘key’ drug(s) to handle your house dust mites allergy)***

🞏 1 drug only, please specify name of medication: /________________________________/

🞏 2 different drugs, please specify name of medications: /______________________________________/

🞏 3 different drugs or more please specify name of medications: /_______________________________

____________________________________________________________/

1. ***Did you take this ‘treatment of reference’:***

🞏 As a cure (during several weeks / months in a row, regardless of the symptoms intensity)

🞏 On demand (during symptoms intensity peaks only)

1. ***Is this ‘treatment of reference’:***

🞏 Prescription drug(s) only

🞏 OTC drug(s) only

🞏 A mix of prescription & OTC drug

1. ***Do you feel like your symptoms have been adequately controlled by these treatments (controlled = absence of symptoms) over the last 12 months?***

☐Not controlled at all ☐ Very slightly controlled ☐ More or less controlled

☐ Well controlled ☐Completely controlled

***Please note on a scale from 0 (not controlled at all) to 10 (completely controlled): ___/ 10***

1. ***Have you ever heard of a desensitisation treatment?***

🞏 Yes 🞏 no

1. **If Q16 = Yes*: Has anyone suggested a desensitisation treatment for your house dust mite allergy over the last 12 months?***

🞏 Yes 🞏 NO

1. **If Q17 = YES*: which doctor suggested this treatment?***

🞏 General Practitioner

🞏 Allergist

🞏 ENT

🞏 Dermatologist

🞏 Pulmonologist

🞏 Paediatrician

🞏 Other, please specify: /________________________________/

1. **If Q17 = YES*: have you accepted to undertake this treatment?***

🞏 Yes

🞏 No

1. **If Q19 = YES*: have you already started this treatment?***

🞏 Yes

🞏 No

1. **If Q20 = YES*: what is the total duration of treatment proposed to you by the physician?***

☐ 1 YEAR
☐ 2 YEARS
☐ 3 YEARS
☐ 4 YEARS
☐ 5 YEARS OR MORE
☐ I DON'T KNOW

1. **If Q20 = YES*: what form of treatment have you been prescribed?***

☐ Injections
☐ Drops
☐ Tablets

1. **If Q19 = NO*: Why did you refuse this desensitisation treatment?*** *(please specify all the reasons for your refusal for each treatment refused)*

/__________________________________________________________________________________________________________________________________________________________________________/

1. **If Q19 = NO*: Are you planning on following a desensitisation treatment one day?***

☐ Yes
☐ No
