# Supplementary figures and images for: “A year-long, fortnightly, observational survey in three European countries of patients with respiratory allergies induced by house dust mites: Methodology, demographics and clinical characteristics”
Source: BMC Pulm Med. 2016 May 23;16:85. doi: 10.1186/s12890-016-0246-9 (PMC4877752; doi:10.1186/s12890-016-0246-9)

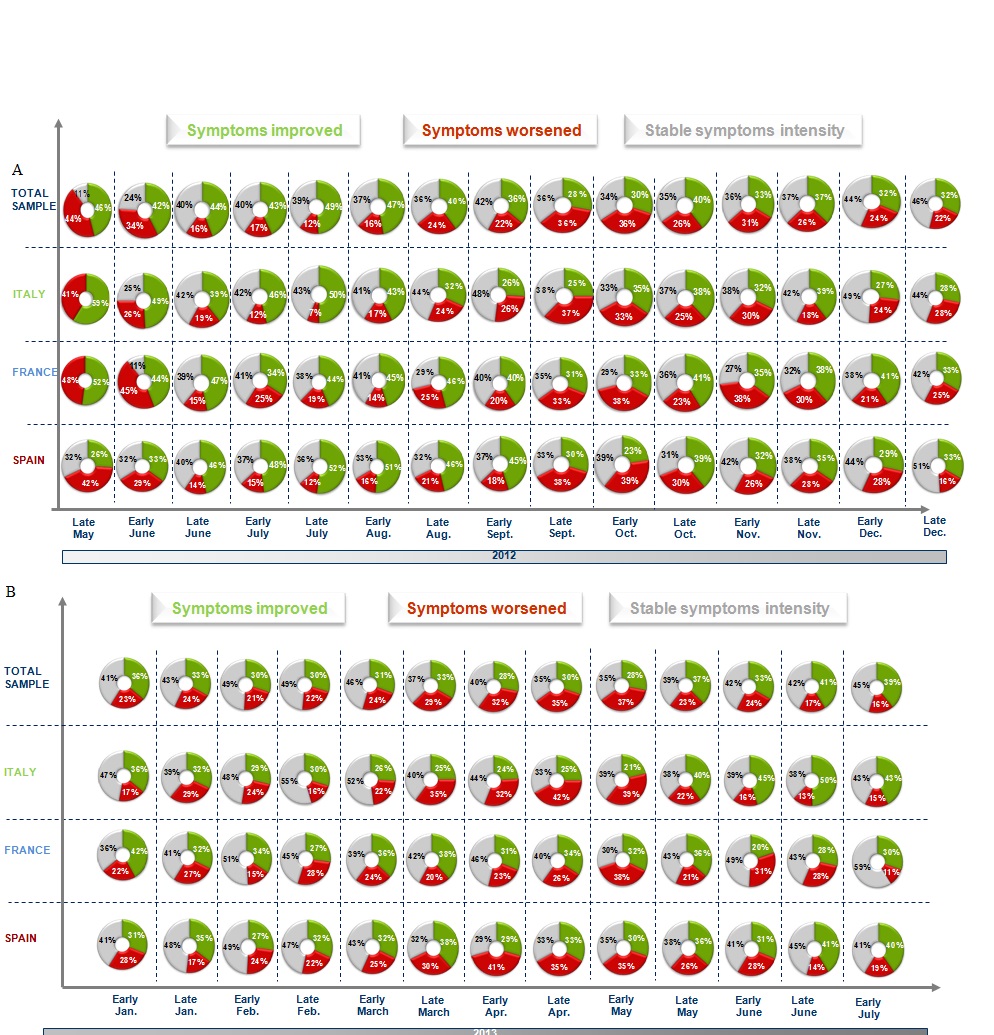

Supplement: Additional file 5: Figure S1. — Whole survey population - late May to December 2012/Whole survey population - January to July 2013. (JPG 413 kb) [file 12890_2016_246_MOESM5_ESM.jpg]

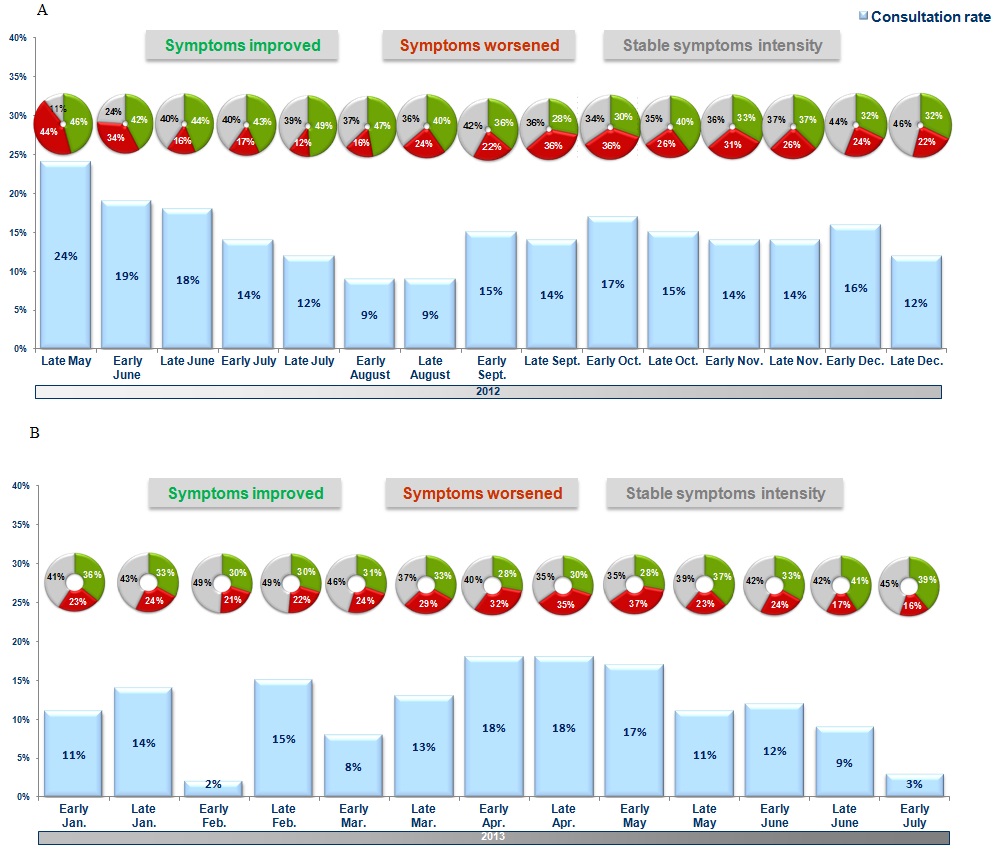

Supplement: Additional file 6: Figure S2. — Late May to December 2012/January to July 2013. (JPG 206 kb) [file 12890_2016_246_MOESM6_ESM.jpg]
